# Comment on Wang et al. Development of a Novel Double Antibody Sandwich ELISA for Quantitative Detection of Porcine Deltacoronavirus Antigen. *Viruses* 2021, *13*, 2403

**DOI:** 10.3390/v14040838

**Published:** 2022-04-18

**Authors:** Ming Li, Chi Zhang, Tianfei Yu

**Affiliations:** 1 College of Computer and Control Engineering, Qiqihar University, Qiqihar 161006, China; fionalee629@163.com (M.L.); 2020935664@qqhru.edu.cn (C.Z.); 2 Heilongjiang Provincial Key Laboratory of Resistance Gene Engineering and Protection of Biodiversity in Cold Areas, College of Life Science and Agriculture and Forestry, Qiqihar University, Qiqihar 161006, China

We were interested in reading an article published by Wang et al. [1] in *Viruses* on 30 November 2021. The authors developed a double-antibody sandwich enzyme-linked immunosorbent assay (DAS-ELISA) for porcine deltacoronavirus (PDCoV) detection using a monoclonal antibody against the PDCoV N protein and an anti-PDCoV rabbit polyclonal antibody. They used kappa analysis to determine the consistency between the DAS-ELISA and reverse transcriptase real-time PCR (RT-qPCR). The kappa value obtained was 0.827, indicating an almost perfect agreement between the two methods.

Although this article has provided valuable information, some substantial points that may cause misinterpretation of the results need to be clarified. Kappa analysis includes Cohen’s and Fleiss’ kappa analyses. Generally, Cohen’s and Fleiss’ kappa analyses are used to analyze intra- and inter-rater agreements, respectively [2]. Cohen’s kappa analysis is suitable for evaluating two raters, whereas Fleiss’ kappa analysis is suitable for evaluating more than two raters. In Cohen’s kappa analysis, the weighted kappa should be used to calculate the agreement in the presence of more than two categories [3]. According to this article, Cohen’s kappa was applicable based on this situation. Cohen’s kappa was calculated as follows:

(1) $$k=\frac{\sum_{i=1}^{n}\left(p_{ii}-p_{i}q_{i}\right)}{1-\sum_{i=1}^{n}p_{i}q_{i}}$$

The value of p and q are the sample frequency.

The authors compared the two detection methods using two types of clinical samples, namely, 205 fecal and 59 intestinal samples. Unlike the authors, we calculated the agreement between the RT-qPCR and DAS-ELISA by using the two types of samples with SPSS 18 statistical package (SPSS 18 Inc., Chicago, IL, USA) software. The kappa values in the fecal and intestinal samples were 0.807 and 0.645, respectively. Furthermore, a simple sum of the data was performed, and the kappa value obtained was 0.781 (Table 1). The three kappa values were significantly different from the authors’ kappa value of 0.827. We would be grateful if the authors could explain their results in detail and clarify the misunderstanding.

Considering the applicability of Cohen’s kappa analysis, we suggest that the kappa values should be calculated in the presence of two or more types of samples. In our opinion, any agreed conclusions must be supported by methodological and statistical methods. We emphasize the importance of rigor and using the correct statistical approach in any scientific publication. Otherwise, misinterpretation cannot be avoided.

## Figures and Tables

**Table 1:** The kappa values for calculating agreement between RT-qPCR and DAS-ELISA.

	DAS-ELISA			
RT-qPCR	Fecal	Positive	Negative	Total
	Positive	73	14	87
	Negative	5	113	118
K = 0.808	Total	78	127	205
	Intestinal	Positive	Negative	Total
	Positive	11	6	17
	Negative	2	40	42
K = 0.645	Total	13	46	59
	Fecal and Intestinal	Positive	Negative	Total
	Positive	84 (73 + 11)	20 (14 + 6)	104
	Negative	7 (5 + 2)	153 (113 + 40)	160
K = 0.781 (K* = 0.827)	Total	91	173	264

Note: The table has been cited from the article published by Wang et al. [1] and undergone moderate modification. K* in the table is the kappa value calculated by Wang et al. [1].
